# Novel Correlation between TGF-β1/-β3 and Hormone Receptors in the Human Corneal Stroma

**DOI:** 10.3390/ijms241713635

**Published:** 2023-09-04

**Authors:** Alexander J. Choi, Brenna S. Hefley, Sarah E. Nicholas, Rebecca L. Cunningham, Dimitrios Karamichos

**Affiliations:** 1 North Texas Eye Research Institute, University of North Texas Health Science Center, Fort Worth, TX 76107, USA; alexander.choi@unthsc.edu (A.J.C.); brenna.hefley@unthsc.edu (B.S.H.); sarah.nicholas@unthsc.edu (S.E.N.); 2 Department of Pharmaceutical Sciences, University of North Texas Health Science Center, Fort Worth, TX 76107, USA; rebecca.cunningham@unthsc.edu; 3 Department of Pharmacology and Neuroscience, University of North Texas Health Science Center, Fort Worth, TX 76107, USA

**Keywords:** cornea, corneal stroma, corneal fibrosis, sex hormone receptors, TGF-β1, TGF-β3, 3D in vitro model, SABV

## Abstract

This study investigated the interplay between transforming growth factor beta (TGF-β1/T1 and TGF-β3/T3), and sex hormone receptors using our 3D in vitro cornea stroma model. Primary human corneal fibroblasts (HCFs) from healthy donors were plated in transwells at 10^6^ cells/well and cultured for four weeks. HCFs were supplemented with stable vitamin C (VitC) and stimulated with T1 or T3. 3D construct proteins were analyzed for the androgen receptor (AR), progesterone receptor (PR), estrogen receptor alpha (ERα) and beta (ERβ), luteinizing hormone receptor (LHR), follicle-stimulating hormone receptor (FSHR), gonadotropin-releasing hormone receptor (GnRHR), KiSS1-derived peptide receptor (KiSS1R/GPR54), and follicle-stimulating hormone subunit beta (FSH-B). In female constructs, T1 significantly upregulated AR, PR, ERα, FSHR, GnRHR, and KiSS1R. In male constructs, T1 significantly downregulated FSHR and FSH-B and significantly upregulated ERα, ERβ, and GnRHR. T3 caused significant upregulation in expressions PR, ERα, ERβ, LHR, FSHR, and GNRHR in female constructs, and significant downregulation of AR, ERα, and FSHR in male constructs. Semi-quantitative Western blot findings present the interplay between sex hormone receptors and TGF-β isoforms in the corneal stroma, which is influenced by sex as a biological variable (SABV). Additional studies are warranted to fully delineate their interactions and signaling mechanisms.

## 1. Introduction

The human cornea, the transparent outer layer of the eye, provides protection to the inner contents of the eye and supplies two-thirds of its refractive power [1,2,3,4,5]. Corneal trauma occurring from injury and/or disease [6,7,8,9,10] can significantly disrupt the corneal structure and homeostasis, leading to scarring and vision impairments [6,10,11,12,13,14]. The complex healing process that is initiated [14,15] post-injury is orchestrated by the corneal stromal resident cells, termed keratocytes, which differentiate into myofibroblasts, proliferate, and migrate into the open wound site [3,9,10,16,17]. This leads to improper extracellular matrix (ECM) deposition, corneal fibrosis, and ultimately impaired vision [3,10,16,17]. Corneal transplantation [18,19,20] remains the most effective treatment to restore the injured cornea. Studies report high success rates of full thickness corneal transplantation (80% to ~96%), with up to 20% [18,21,22] post-operative (post-op) complications. Other complications include donor cornea rejection, cataract formation, and vascularization [18,23,24]. Sex as a biological variable (SABV) in the context of corneal fibrosis and its treatment(s) is largely unexplored.

The presence of sex hormones in the cornea has been previously reported [25,26,27,28,29], as well as in the aqueous and vitreous humors of the eye [26,27]. A study conducted by Suzuki et al. [29] reported the presence of estrogen receptor-α (ERα), estrogen receptor-β (ERβ), and the progesterone receptor (PR) in the human cornea, suggesting that sex hormones affect the biological functions in the cornea. We recently reported the effects of estrone and estriol on the human corneal stroma ECM, and highlighted the differences between healthy and keratoconic stromal cell origin [28]. Other receptors, such as the luteinizing hormone receptor (LHR) and follicle-stimulating hormone receptor (FSHR), are also expressed by human corneal stroma [27], suggesting that the human cornea may be able to produce hormones in situ as well as respond to hormonal imbalances, thereby influencing localized cellular/molecular signaling. Despite the presence of sex hormone receptors in the human corneal [5,25,27,28,29], their role in corneal homeostasis remains unclear [26].

Transforming growth factor-beta (TGF-β) isoforms have been correlated with fibrosis in numerous organs and tissues [9,30,31,32,33], as well as the human cornea [34,35,36,37]. TGF-β is able to modulate tissue/cell functions [38] through its three main isoforms: TGF-β1 (T1), TGF-β2 (T2), and TGF-β3 (T3). Briefly, T1 was the first member to be identified in the TGF-β family [39]. In the cornea, it was discovered to be produced by several cell types, including corneal epithelial cells [30]. T1 also drives stromal keratocyte differentiation into active myofibroblasts, leading to ECM remodeling at the wounded site [40]. Meanwhile, T3 is thought to be an anti-fibrotic modulator, despite sharing highly similar peptide structures with T1 and T2 (70–80% homologies) [41,42,43]. To-date, T1 and T2 are known to induce corneal fibrosis [9,11,13,39,44,45], while T3 is known for its anti-fibrotic impact [9,11,13,26,31,39,44,45,46,47,48,49,50]. Outside of fibrosis, a deficiency in TGF-β has been associated with numerous pathological conditions, such as autoimmune diseases [51], atherosclerosis [52], and defective wound repair [38,53]. An overexpression of TGF-β has been linked to immunopathologies [54,55], including cancer [38,56].

The objective of the current study was to investigate the novel interactions between corneal stroma hormone receptors and T1/T3 isoforms, using an established 3D self-assembled ECM in vitro model. Further, we highlight the potential SABV impact in the system described.

## 2. Results

### 2.1. Androgen Receptor (AR) and Progesterone Receptor (PR)

**Figure 1:**
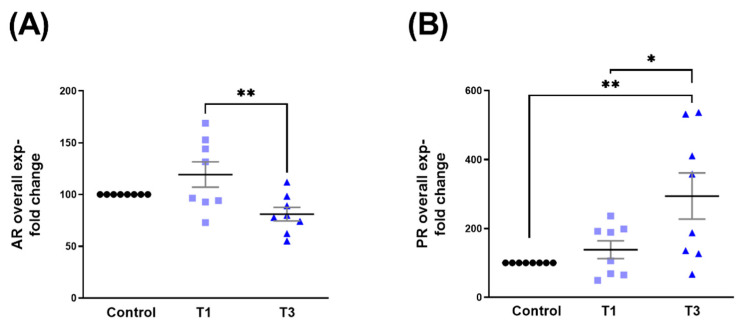
AR and PR protein expression with control, T1, and T3 stimulation on 3D HCF constructs. (**A**) Overall AR expression with T1 and T3 stimulation when compared to controls. (**B**) Overall PR expression with T1 and T3 stimulation when compared to controls. * = *p* < 0.05; ** = *p* < 0.01.

Protein expressions for AR and PR were investigated in 3D human corneal fibroblast (HCF) constructs stimulated with T1 or T3. Overall AR expression was significantly upregulated with T1 compared to T3, but not compared to controls (Figure 1A). Overall PR expression was significantly upregulated with both T1 and T3 stimulation, when compared to controls (Figure 1B). No differences were observed between T1 and T3 (Figure 1B).

**Figure 2:**
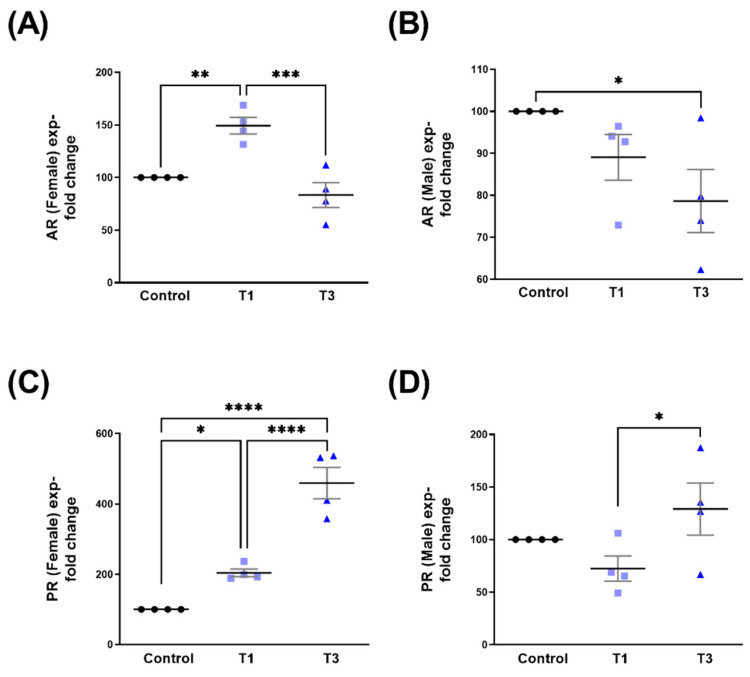
AR and PR protein expression with control, T1, and T3 stimulation between HCF-Fs and HCF-Ms. (**A**) HCF-Fs AR expression for T1 and T3 stimulation when compared to controls. (**B**) HCF-Ms AR expression stimulated with T1 and T3 when compared to controls. (**C**) HCF-Fs PR expression with T1 and T3 stimulation when compared to control. (**D**) HCF-Ms PR expression with T1 and T3 stimulation when compared to controls. * = *p* < 0.05; ** = *p* < 0.01; *** = *p* < 0.001; **** = *p* < 0.0001.

Related to SABV, stimulation with T1 in female 3D HCFs (HCF-Fs) led to the significant upregulation of AR when compared to both controls and T3 (Figure 2A). In male 3D HCFs (HCF-Ms), T3 led to the significant downregulation of AR expression compared to controls (Figure 2B). PR expression in HCF-Fs was significantly upregulated in both T1 and T3, when compared to controls. The PR in HCF-Fs stimulated with T3 was significantly higher when compared to T1 (Figure 2C). Interestingly PR expression in HCF-Ms was not impacted by T1 or T3, compared to controls (Figure 2D). PR expression, however, was significantly upregulated with T3, as compared to T1 (Figure 2D). Corresponding Western blot images are shown in Figure S1.

### 2.2. Estrogen Receptor Alpha (ERα) and Estrogen Receptor Beta (ERβ)

**Figure 3:**
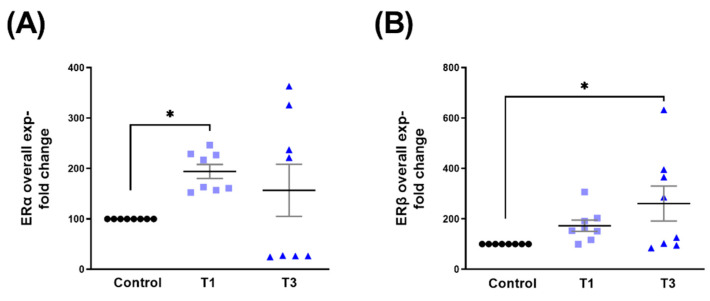
Overall ERα and ERβ protein expression with control, T1, and T3 stimulation on 3D HCF constructs. (**A**) Overall ERα expression with T1 and T3 stimulation when compared to controls. (**B**) Overall ERβ expression with T1 and T3 stimulation when compared to controls. * = *p* < 0.05.

The overall expression of ERα was significantly upregulated with T1 (Figure 3A), whereas ERβ expression was significantly upregulated with T3 only (Figure 3B), when compared to controls.

**Figure 4:**
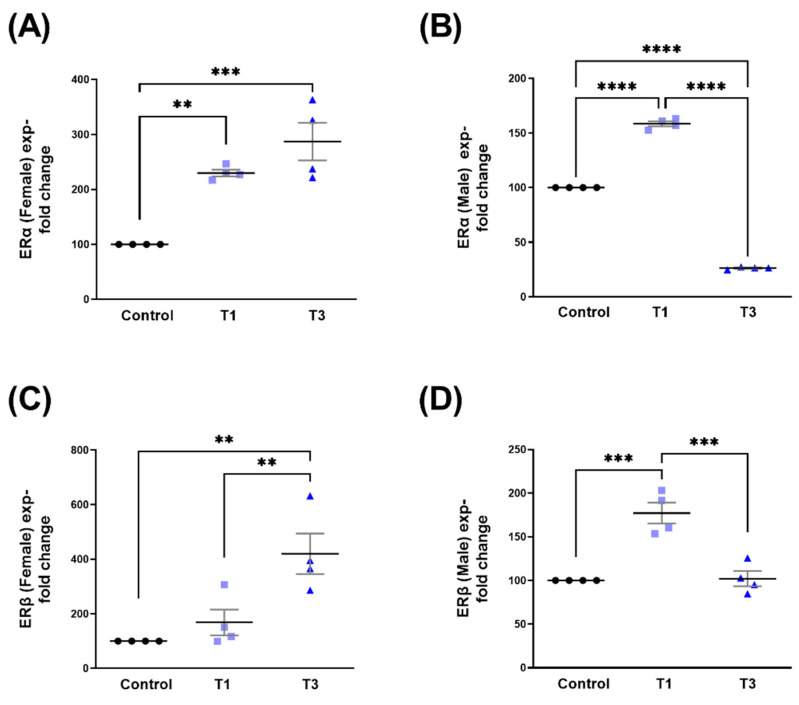
ERα and ERβ protein expression with control, T1, and T3 stimulation between HCF-Fs and HCF-Ms. (**A**) ERα expression change with T1 and T3 stimulation when compared to controls for HCF-Fs. (**B**) ERα expression stimulated with T1 and T3 when compared to controls for HCF-Ms. (**C**) ERβ expression with T1 and T3 stimulation compared to controls for HCF-Fs. (**D**) ERβ expression with T1 and T3 stimulation compared to controls for HCF-Ms. ** = *p* < 0.01; *** = *p* < 0.001; **** = *p* < 0.0001.

SABV data stratification showed the upregulation of ERα expression in HCF-Fs by both T1 and T3 when compared to controls (Figure 4A). In HCF-Ms, ERα was significantly upregulated with T1 but downregulated with T3 (Figure 4B). Furthermore, HCF-Ms ERα expression was significantly downregulated by T3 when compared to T1 (Figure 4B). Notably, in HCF-Fs, ERβ modulation by T1/T3 was very similar to ERα (Figure 4A), showing significant upregulation by T1 and T3 (Figure 4C). In HCF-Ms, ERβ expression was significantly upregulated by T1 but not affected by T3 (Figure 4D). Corresponding Western blot images are shown in Figure S2.

### 2.3. Luteinizing Hormone Receptor (LHR), Gonadotropin-Releasing Hormone Receptor (GnRHR), and Follicle-Stimulating Hormone Receptor (FSHR)

**Figure 5:**
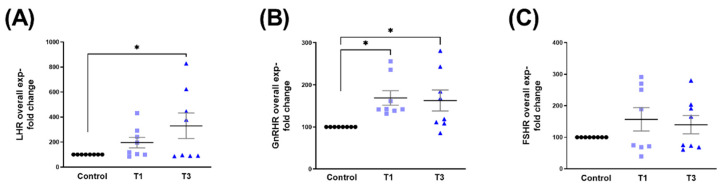
Overall LHR, GnRHR, and FSHR protein expressions with control, T1, and T3 stimulation on 3D HCF constructs. (**A**) Overall LHR expression with T1 and T3 stimulation when compared to controls. (**B**) Overall GnRHR expression with T1 and T3 stimulation compared to controls. (**C**) Overall FSHR expression when stimulated with T1 and T3 stimulation when compared to controls. * = *p* < 0.05.

T3 led to the significant upregulation of the overall LHR expression compared to controls (Figure 5A). Overall GnRHR expression was significantly upregulated by both T1 and T3, when compared to controls (Figure 5B), whereas FSHR expression was not impacted by either one of the TGF-β isoforms (Figure 5C).

**Figure 6:**
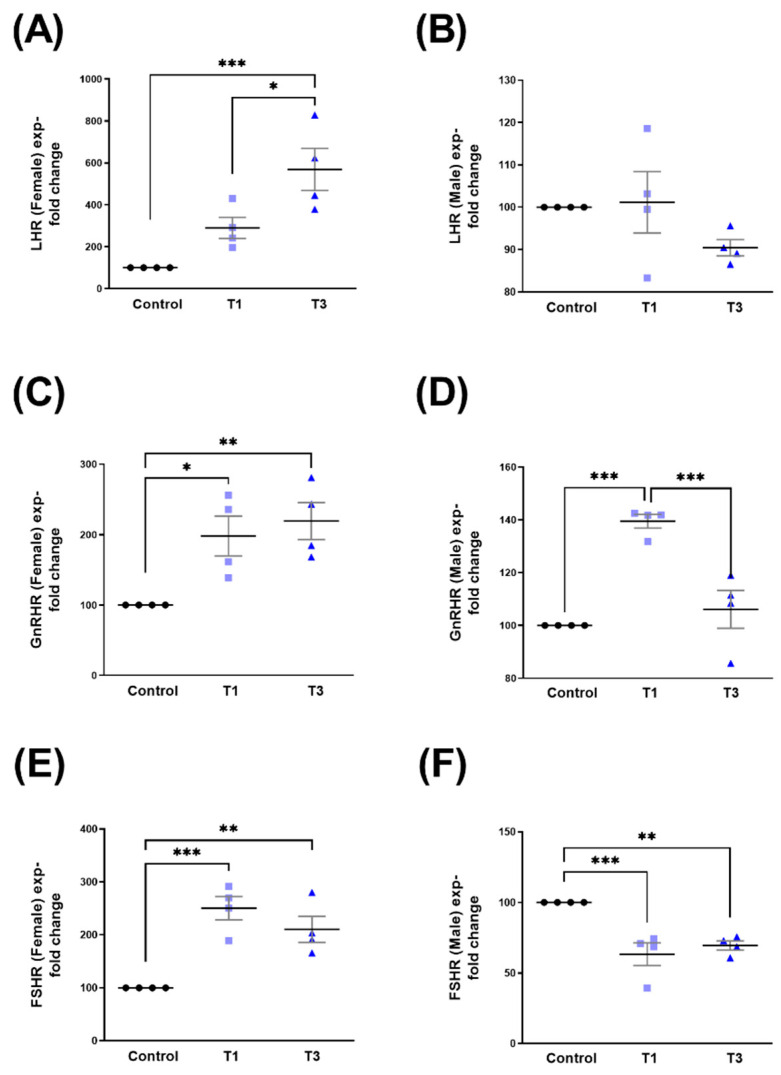
LHR, GnRHR, and FSHR protein expression with control, T1, and T3 stimulation between HCF-Fs and HCF-Ms. (**A**) LHR expression with T1 and T3 stimulation compared to controls for HCF-Fs. (**B**) LHR expression when stimulated with T1 and T3 compared to controls for HCF-Ms. (**C**) GnRHR expression when stimulated with T1 and T3 when compared to controls for HCF-Fs. (**D**) GnRHR expression when stimulated with T1 and T3 when compared to controls for HCF-Ms. (**E**) FSHR expression when stimulated with T1 and T3 compared to controls for HCF-Fs. (**F**) FSHR expression when stimulated with T1 and T3 when compared to controls for HCF-Ms. * = *p* < 0.05; ** = *p* < 0.01; *** = *p* < 0.001.

Significant upregulation was observed in LHR, GnRHR, and FSHR in HCF-Fs stimulated with both T1 and T3 (Figure 6A, Figure 6C, and Figure 6E, respectively). In HCF-Ms, LHR was not modulated by T1 or T3 (Figure 6B), whereas GnRHR was significantly upregulated by T1, but downregulated by T3 when compared to its controls (Figure 6D). FSHR expression in HCF-Ms showed significant downregulation when stimulated with T1 and T3, when compared to controls (Figure 6F). Corresponding Western blot images are shown in Figure S3.

### 2.4. Thyrotropin-Releasing Hormone Receptor (TRHR) and KiSS1-Derived Peptide Receptor (KISS1R/GPR54)

**Figure 7:**
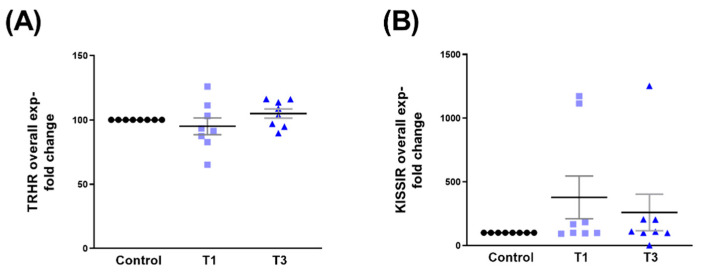
Overall TRHR and KISS1R protein expressions with control, T1, and T3 stimulation on 3D HCF constructs. (**A**) TRHR expression when stimulated with T1 and T3 stimulation compared to the controls. (**B**) KISS1R expression with T1 and T3 stimulation when compared to controls.

The overall expressions of TRHR (Figure 7A) and KISS1R (Figure 7B) were not modulated by either T1 or T3.

**Figure 8:**
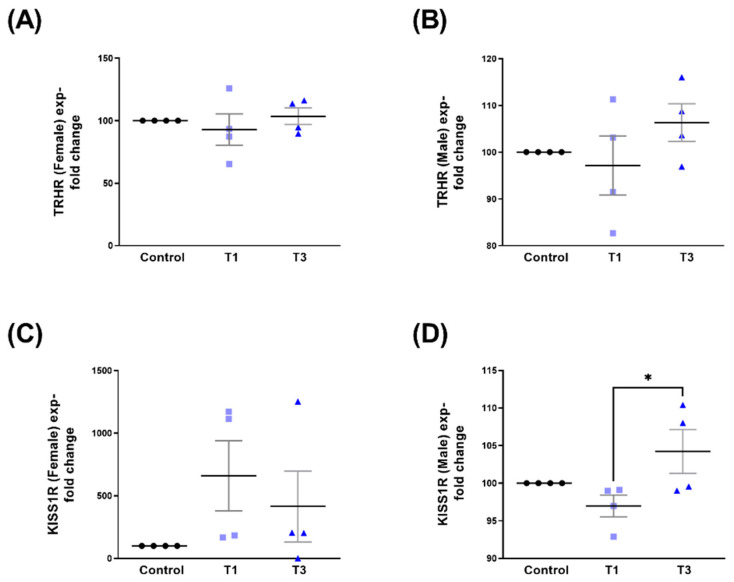
TRHR and KISS1R protein expressions with control, T1, and T3 stimulation between HCF-Fs and HCF-Ms. (**A**) TRHR expression when stimulated with T1 and T3 compared to controls for HCF-Fs. (**B**) TRHR expression when stimulated with T1 and T3 compared to controls for HCF-Ms. (**C**) KISS1R expression when stimulated with T1 and T3 compared to controls for HCF-Fs. (**D**) KISS1R expression when stimulated with T1 and T3 compared to controls for HCF-Ms. * = *p* < 0.05.

When data were stratified based on sex, both TRHR (Figure 8A,B) and KISS1R (Figure 8C,D) showed no significant changes following T1 or T3 stimulation, when compared to controls. The significant upregulation of KISS1R HCF-Ms following T3 stimulation was, however, observed when compared to T1 (Figure 8D). Corresponding Western blot images are shown in Figure S4.

### 2.5. G Protein Subunit Alpha Q (GNAQ), G Protein Subunit Alpha 11 (GNA-11), and G Protein Alpha Stimulating (GNAS)

**Figure 9:**
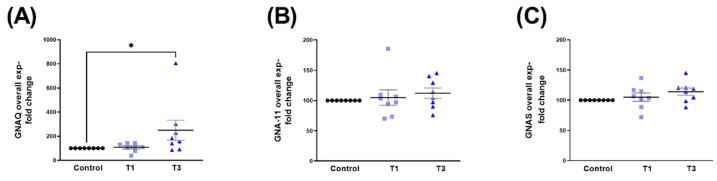
Overall GNAQ, GNA11, and GNAS protein expressions with control, T1, and T3 stimulation on 3D HCF constructs. (**A**) Overall GNAQ expression with T1 and T3 stimulation compared to controls. (**B**) GNA11 overall expression stimulated with T1 and T3 compared to controls. (**C**) Overall GNAS expression when stimulated with T1 and T3 compared to controls. * = *p* < 0.05.

The overall expression of GNAQ was significantly upregulated with T3 when compared to controls (Figure 9A). Both GNA11 and GNAS showed no changes when stimulated with T1 or T3 (Figure 9B and Figure 9C, respectively).

**Figure 10:**
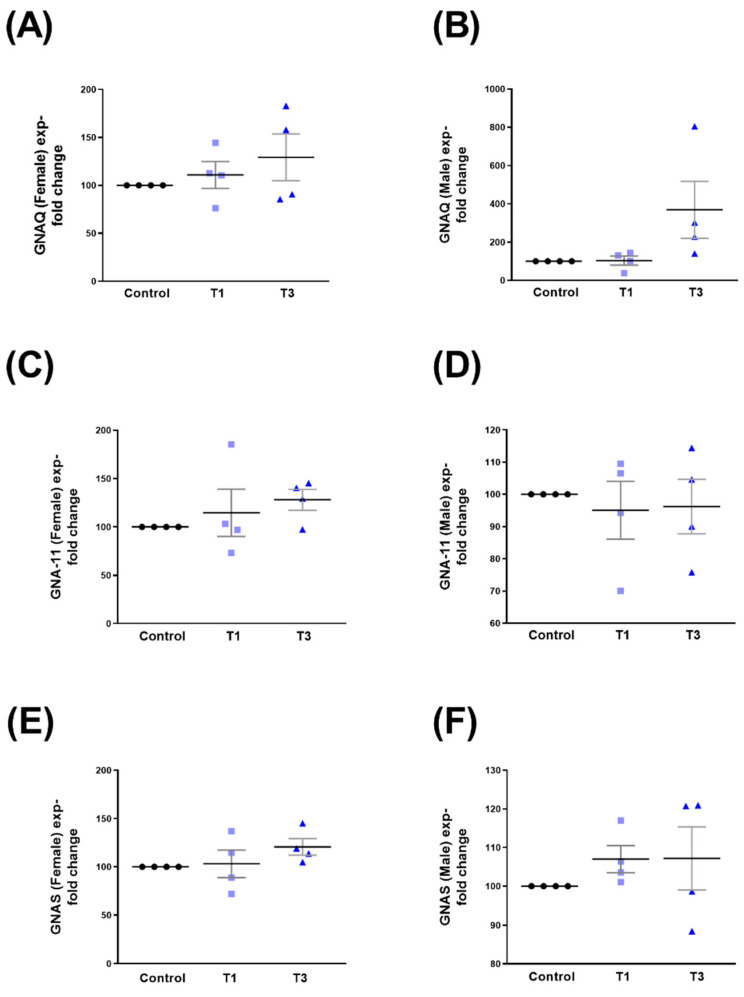
GNAQ, GNA11, and GNAS protein expression with control, T1, and T3 stimulation between HCF-Fs and HCF-Ms. (**A**) GNAQ expression in HCF-Fs when stimulated with T1 and T3 compared to controls. (**B**) GNAQ expression in HCF-Ms when stimulated with T1 and T3 compared to controls. (**C**) GNA11 expression in HCF-Fs when stimulated with T1 and T3 compared to controls. (**D**) GNA11 expression when stimulated with T1 and T3 compared to controls in HCF-Ms. (**E**) GNAS expression stimulated with T1 and T3 when compared to controls for HCF-Fs. (**F**) GNAS expression for HCF-Ms when stimulated with T1 and T3 compared to controls.

We observed no significant changes in either sex (HCF-Ms and HCF-Fs) in the expressions of GNAQ (Figure 10A,B), GNA11 (Figure 10C,D), and GNAS (Figure 10E,F), following T1/T3 stimulation. Corresponding Western blot images are shown in Figure S5.

### 2.6. Follicle-Stimulating Hormone Subunit Beta (FSH-B)

**Figure 11:**
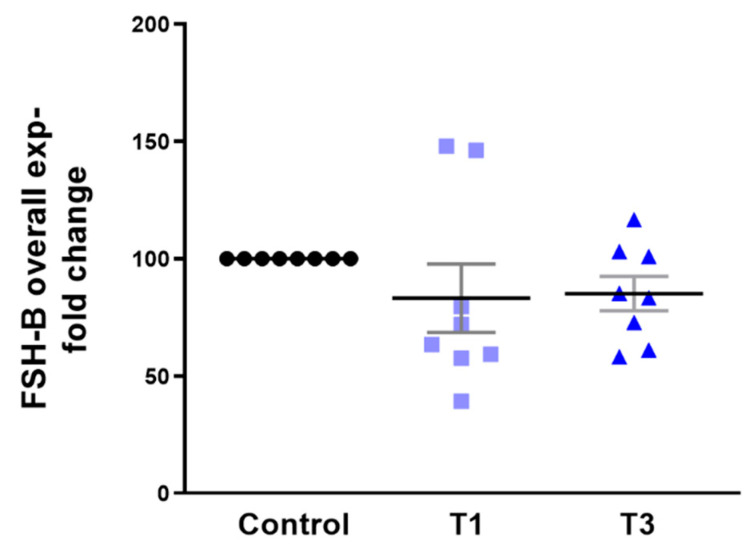
Overall FSH-B protein expression with T1 and T3 stimulation on 3D HCF constructs when compared to controls.

Overall FSH-B expression showed no significant changes with any of the stimulations tested here (Figure 11).

**Figure 12:**
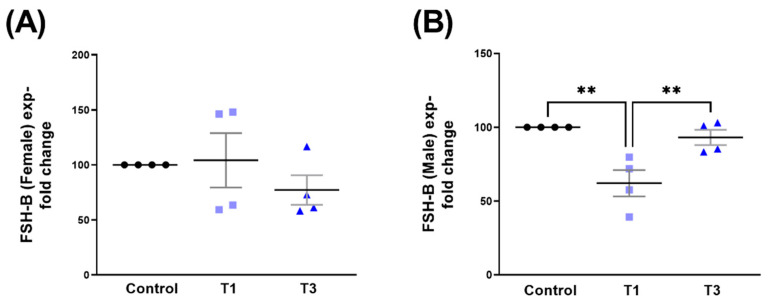
FSH-B protein expression with control, T1, and T3 stimulation between HCF-Fs and HCF-Ms. (**A**) FSH-B expression in HCF-Fs when stimulated with T1 and T3 compared to controls. (**B**) FSH-B expressed in HCF-Ms with T1 and T3 stimulation compared to controls. ** = *p* < 0.01.

FSH-B expression was unaffected in HCF-Fs (Figure 12A). However, in HCF-Ms, stimulation with T1 led to the significant downregulation of FSH-B when compared to both controls and T3 (Figure 12B). Corresponding Western blot images are shown in Figure S6.

## 3. Discussion

TFG-β is a major regulator of numerous cellular processes [57,58,59,60,61]. Disruption in the TGF-β signaling pathway can lead to connective tissue disorders, cancer, and/or fibrosis [57]. There are currently 33 known human TGF-β family polypeptides, including the three TGF-β isoforms: T1, T2, and T3 [49,57,62]. TGF-β is found throughout the body, including in the human cornea [9].

Recent studies have shown that inhibiting T1 can reduce fibrosis in the cornea [63,64,65]. Chang et al. investigated a potentially useful anti-fibrotic therapy in the cornea using hypercapnic acidosis [63]. The authors observed that when the cells were grown under hypercapnic acidosis conditions, alpha smooth muscle actin (α-SMA), collagen gel contraction, and T1 induced corneal fibroblast migration were suppressed, demonstrating the potential of hypercapnic acidosis as an anti-fibrotic therapy [63]. Zahir-Jouzdani et al. investigated the utilization of nanoparticles loaded with anti-fibrotic T1 siRNA as a potential topical delivery system [64]. Their findings indicated that the delivery system was able to suppress T1 platelet-derived growth factor (PDGF) genes and ECM deposition in isolated human corneal fibroblasts [64]. The nanoparticles were also able to inhibit α-SMA and the proliferation and transformation of fibroblasts into myofibroblasts [64].

T3 inhibits fibrotic markers, tissue fibrosis, and scar formation [15]. Karamichos et al. examined the effects of T1 and T3 [15] and observed increased expressions of type III collagen and α-SMA in 3D HCF constructs treated with T1, with significant downregulation in constructs treated with T3 [15]. Their findings correlated with previous data [48], demonstrating the anti-fibrotic effects of T3 treatment [15,48]. Guo et al. stimulated HCFs with T1 or T3 and harvested the cells after 4 h or 3 days [66]. The authors found that T3 upregulates the Suppressor of Mothers against Decapentaplegic 7 (Smad7) and thrombospondin-1 (THBS1), which promoted a non-fibrotic ECM in their 3D cell culture model [66]. The maintenance of corneal transparency is a complex and precise process. Corneal wound healing requires precise ECM secretion, deposition, and organization by the myofibroblasts [26].

SABV in the context of corneal wound healing is severely understudied. Tripathi et al. investigated sex-based differences in corneal wound healing in New Zealand White rabbits [49], where no sex-based changes were observed in the mRNA or protein levels of α-SMA, fibronectin (FN), Collagen-I (Col-I), and T1 [49], following topical alkali burns. Others have looked into sex-based differences, such as sex hormones, in various species [29,67,68]. Some sex hormones and their receptors can be found in both sexes, but can vary in levels depending on the sex [69,70,71]. Estrogen and its receptors have been studied in both sexes, even though it was traditionally considered a female hormone [72,73]. Suzuki et al. investigated the existence of estrogen receptors in the human cornea and observed the expression of ERα and ERβ [29]. Additionally, Wickham et al. examined the mRNAs of sex hormone receptors from rabbit eyes and found sex- and tissue-specific differences [67]. Other studies suggest that sex hormone changes such as menopause, menstrual cycles, and pregnancy can likely influence the corneal stroma [26,67,74,75,76]. During the menstrual cycle, hormone levels are known to fluctuate, including estrogen, which increases and decreases twice throughout the cycle [77]. Estrogen rises in the mid-follicular phase and during the mid-luteal phase. Estrogen decreases after ovulation and towards the end of the menstrual cycle. When estrogen binds with its receptor, it can regulate different pathways, including nuclear factor kappa B (NF-κB) [78], c-Jun N-terminal kinase (JNK) [78], and cytokines, such as TGF-β [79]. Kanda et al. found that Smad3 may be involved in androgen-induced mice wound healing, mediating signals from TGF-β, which does not occur in castrated Smad3 null mice [68]. Conversely, estrogen stimulates Smad2/3 protein degradation, inhibiting TGF-β signaling [80]. In females, estrogen, progesterone, and androgens are able to interact with most TGF-β superfamily members [80]. In males, the complex network of BMP/TGF-β signaling is essential in their reproductive biology [80].

In our study, we investigated sex hormone expressions when treated with T1 and T3 using healthy corneal stromal cells (male and female donors). The overall objective was to delineate the role of sex hormones and corneal fibrosis. Throughout our findings, we identified higher expressions in many of the sex hormone receptors with T1 and T3 in HCF-F, but found mostly suppressed expressions with T1 and T3 in HCF-M. The activation of male sex hormone expressions (AR) in females and the activation of female sex hormone expressions (ERα and ERβ) in males when treated with T1 indicate a complex regulation of sex-specific hormones in the corneal fibrosis cascade. The present study is limited by the fact that only one male and one female donor were examined. Thus, future studies are warranted in order to fully understand the modulation of corneal sex hormone receptors in the context of SABV.

In addition to looking into sex-specific hormones, we also investigated Guanine nucleotide-binding proteins (G-proteins) [81,82,83]. Numerous studies have identified G-proteins in different parts of the eye, but very little is known about the cornea [81,82,83,84,85,86,87]. The actions of G-proteins enable the process of channeling signals through the cell surface to the intracellular effectors [88]. During this process, the G-proteins transfer signals from G-protein coupled receptors (GPCR), allowing the binding of agonists, which induces the GPCRs into an active conformational state [88,89]. GGPCRs are involved heavily in human physiology and behavior, which includes hormones and neurotransmitters [89,90]. While the term “G-protein” is used in different formats, there are three G-protein subunits (α, β, and γ) that are necessary in the interaction between the protein and associated receptors [89,90]. However, due to the tight association between the β and γ subunits, they are considered as one functional unit, making the known two functional subunits labeled as Gα and Gβγ [89,90]. With the GPCRs’ development due to the active conformation, the actions involving the G-protein signaling process increases [89]. While G-proteins are involved in the regulation of many processes, such as protein synthesis and the transport process [91], signaling pathways that are associated with G-proteins are usually involved in cellular responses, including hormones, antigens, and variations in extracellular matrix and cell-cell contacts [88]. Our studies revealed that the expression of the G-proteins and receptors surprisingly showed minimal involvement in the linkage across the GnRHR G-protein subunit family. However, between the correlations of the GnRHR subunit family, GnRHR and GNA-11 were activated with T3 stimulation in females, while KISS1R and FSHB were activated by T3 compared to T1 in males.

## 4. Materials and Methods

### 4.1. Ethical Consent

Cadaveric human corneas without a history of ocular or systemic disease were obtained from the National Disease Research Interchange (NDRI; Philadelphia, PA, USA) and de-identified prior to processing and analysis. The North Texas Regional Institutional Review Board (#2020-030) reviewed and approved all studies herein. All research conducted adhered to the tenets of the Declaration of Helsinki.

### 4.2. Cell Isolation, Cell Cultures, and ECM Assembly

Primary HCFs were isolated from a healthy 65-year-old male and a healthy 88-year-old female donor for this study. Briefly, the corneal epithelium and endothelium were scraped off from the corneas using a razor blade. The corneal stroma was then cut into ~2 × 2 mm pieces and placed in T25 flasks, where they were allowed to adhere. The explants were then cultured with complete media (Eagle’s Minimum Essential Medium (EMEM; CORNING, Corning, NY, USA) containing 10% of fetal bovine serum (FBS; Atlanta Biologicals; Flowery Branch, GA, USA) and 1% of Antibiotic-Antimycotic (AA; Life Technologies; Grand Island, NY, USA)). All explants were grown to 80% confluence at 37 °C with 5% of CO_2_ before further sub-culturing [92].

Three-dimensional constructs were generated by seeding HCFs in six-well plates with polycarbonate inserts (CELLTREAT Scientific Products; Pepperell, MA, USA) at a density of 1 × 10^6^ cells/well, as previously described [28,92]. All constructs were cultured in complete EMEM with 10% FBS and 1% AA, supplemented by 0.5 mM of stable vitamin C (VitC; 2-O-α-D-glucopyranosyl-L ascorbic acid; Sigma, St. Louis, MO, USA) for 4 weeks. During the 4 weeks in culture, all constructs were given fresh media every other day. As previously optimized by our group, T1 and T3 were used at a concentration of 0.1 ng/mL [46,92,93,94]. The treatment groups were as follows: Controls: complete media + VitC-only; T1 group: complete media + VitC + 0.1 ng/mL T1 (R&D Systems; Minneapolis, MN, USA); and T3 group: complete media + VitC + 0.1 ng/mL T3 (R&D Systems; Minneapolis, MN, USA).

### 4.3. Protein Extraction and Quantification

Protein extraction was performed on all constructs at 4 weeks, as previously described [28,95]. Briefly, culture media were removed and constructs were washed twice with cold 1X Phosphate Buffered Saline (PBS). Constructs were gently scraped from the polycarbonate membranes and suspended in 1X immunoprecipitation buffer (50 mM Tris, pH 8, 150 mM NaCl, 1% Triton X-100, 0.1% SDS, 1% sodium deoxycholate) (Abcam, Cambridge, MA, USA) + 1% Proteinase Inhibitor (PI) cocktail (Sigma; St. Louis, MO, USA), and incubated for 30 min at 4 °C. The samples were then centrifuged for 15 min at 12,000 RPM at 4 °C. The Pierce BCA protein assay kit (Pierce™ Bovine Serum Albumin standards (23208; ThermoFisher Scientific, Waltham, MA, USA)) was used to perform protein quantification. A BioTek EPOCH2 microplate reader (BioTek; Winooski, VT, USA) was utilized for absorbance measurements and calculations [28].

### 4.4. Western Blot

All samples were normalized to equal protein concentrations before denaturing and being added to the Precast Novex 4–20% Tris Glycine Mini Gels (Life Technologies; Carlsbad, CA, USA) for gel electrophoresis. The gels were transferred using iBlot2 Nitrocellulose transfer stacks and incubated at room temperature in a fluorescence blocking solution for 1 h [28]. The blocking solution was then removed and the membranes were incubated overnight with rocking at 4 °C, with the following primary antibodies: Rabbit Polyclonal to GnRHR (ab183079, Abcam, Cambridge, MA, USA) at 1:500, Rabbit Polyclonal to LHR (ab125214, Abcam, Cambridge, MA, USA) at 1:250, Rabbit Polyclonal to FSHR (ab75200, Abcam, Cambridge, MA, USA) at 1:250, Rabbit Polyclonal to AR (ab3510, Abcam, Cambridge, MA, USA) at 1:500, Rabbit Polyclonal to PR (ab191138, Abcam, Cambridge, MA, USA) at 1:500, Rabbit Polyclonal to ERα (ab75635, Abcam, Cambridge, MA, USA) at 1:500, Rabbit Polyclonal to ERβ (ab3576, Abcam, Cambridge, MA, USA) at 1:500, Goat Polyclonal to GNAS (ab101736, Abcam, Cambridge, MA, USA) at 1:500, Rabbit Polyclonal to TRHR (ab72179, Abcam, Cambridge, MA, USA) at 1:250, Rabbit Monoclonal to FSH-B (ab150425, Abcam, Cambridge, MA, USA) at 1:500, Mouse Monoclonal to GNAQ (H00002776-M04, ThermoFisher Scientific, Waltham, MA, USA) at 1:250, Rabbit Polyclonal to KISS1R/GPR54 (NBP2-16724, ThermoFisher Scientific, Waltham, MA, USA) at 1:500, Mouse Monoclonal to GAPDH (ab184578, Abcam, Cambridge, MA, USA) at 1:1000, and Rabbit Polyclonal at GNA11 (PA5-76678, ThermoFisher Scientific, Waltham, MA, USA) at 1:250, conjugated with Biotium CF^®^647 (92218,Thermo Scientific; Waltham, MA, USA). The membranes were washed three times with Tris Buffered Saline (Thermo Fisher Scientific; Waltham, MA USA) and Tween 20 (Sigma; St. Louis, MO, USA) (TBST) and incubated at room temperature with their respective secondary antibodies (AlexaFluor 488 [a32731TR, ThermoFisher Scientific, Waltham, MA, USA], AlexaFluor 568 [ab133273, Abcam, Cambridge, MA, USA], AlexaFluor 647 [a331571, ThermoFisher Scientific, Waltham, MA, USA], AlexaFluor 680 [ab175776, Abcam, Cambridge, MA, USA], and AlexaFluor 750 [ab175738, Abcam, Cambridge, MA, USA]) for 1 h at room temperature. The membranes were washed three times with TBST, imaged using iBright 1500 (Invitrogen; Thermo Fisher Scientific, Waltham, MA, USA), and analyzed using iBright Analysis 5.0.1 Software (Invitrogen; Thermo Fisher Scientific, Waltham, MA, USA). All data were normalized to the GAPDH housekeeping (Figure S7) values and the averages were plotted (mean ± SEM). Representative Western blot images are included in the Supplementary Materials.

### 4.5. Statistical Analysis

All experiments were repeated at least three times and statistical analyses were performed using GraphPad Prism 9.3.0 software. Significance was assessed by one-way ANOVA where *p* < 0.05 was considered statistically significant.

## 5. Conclusions

These observations have revealed a link between sex-dependent regulations of G-proteins and sex hormone receptors in the development of corneal fibrosis. These data are novel and could provide novel diagnostic opportunities and therapeutic targets that could ultimately be used for the treatment of corneal fibrosis. Future in vivo studies are warranted in order to validate these targets before further development. The role of G-proteins and sex hormone-related signaling cascades could indeed provide invaluable diagnostic insights into sex-driven corneal fibrogenesis.

## Data Availability

The data presented in this study are available upon request from the corresponding author.

## Supplementary Materials

The supporting information can be downloaded at: https://www.mdpi.com/article/10.3390/ijms241713635/s1.
