# Supplementary material for: Novel Correlation between TGF-β1/-β3 and Hormone Receptors in the Human Corneal Stroma
Source: Int J Mol Sci. 2023 Sep 4;24(17):13635. doi: 10.3390/ijms241713635 (PMC10487450; doi:10.3390/ijms241713635)
Supplement: Supplementary file 1 [file ijms-24-13635-s001.zip › ijms-2533493-supplementary.pdf]

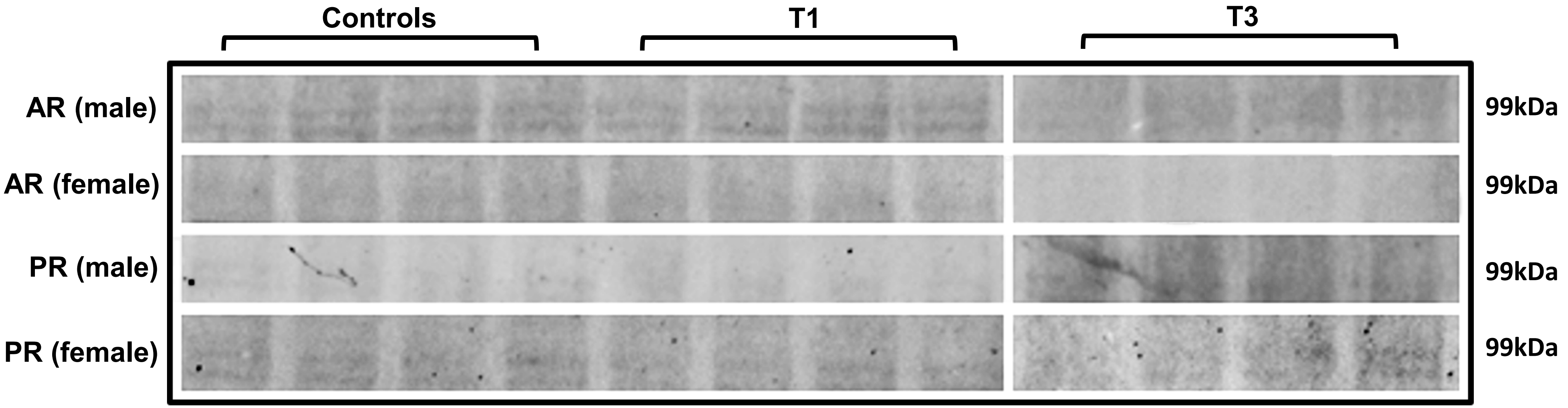

Supplemental Figure S1: Representative Western Blot images of AR and PR protein expression with control, T1, and T3 stimulation between HCF-Fs and HCF-Ms.

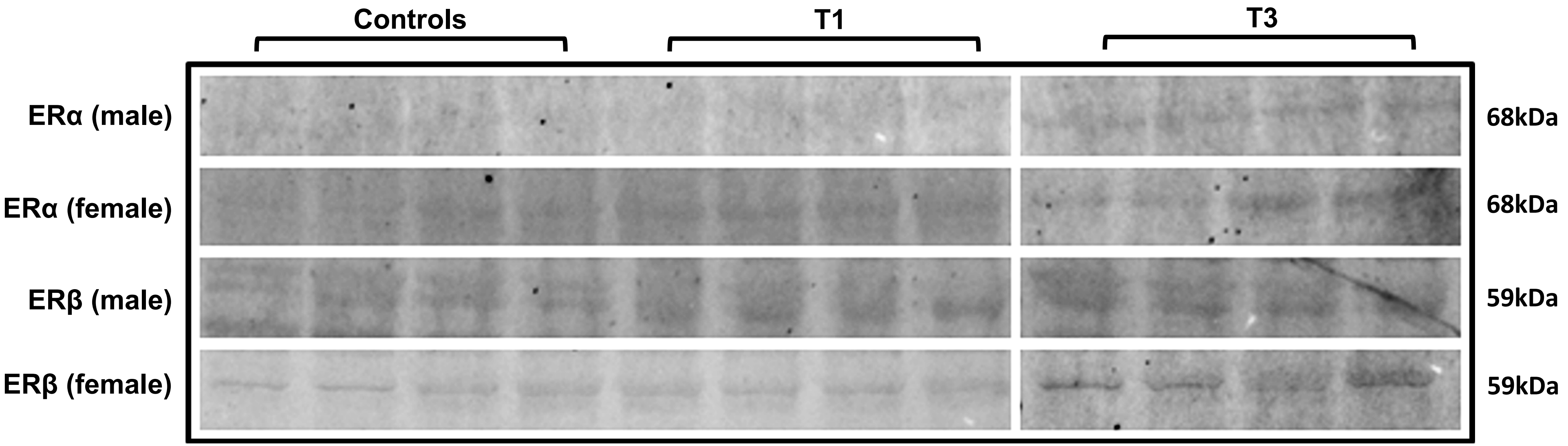

Supplemental Figure S2: Representative Western Blot images of ERα and ERβ protein expression with control, T1, and T3 stimulation between HCF-Fs and HCF-Ms.

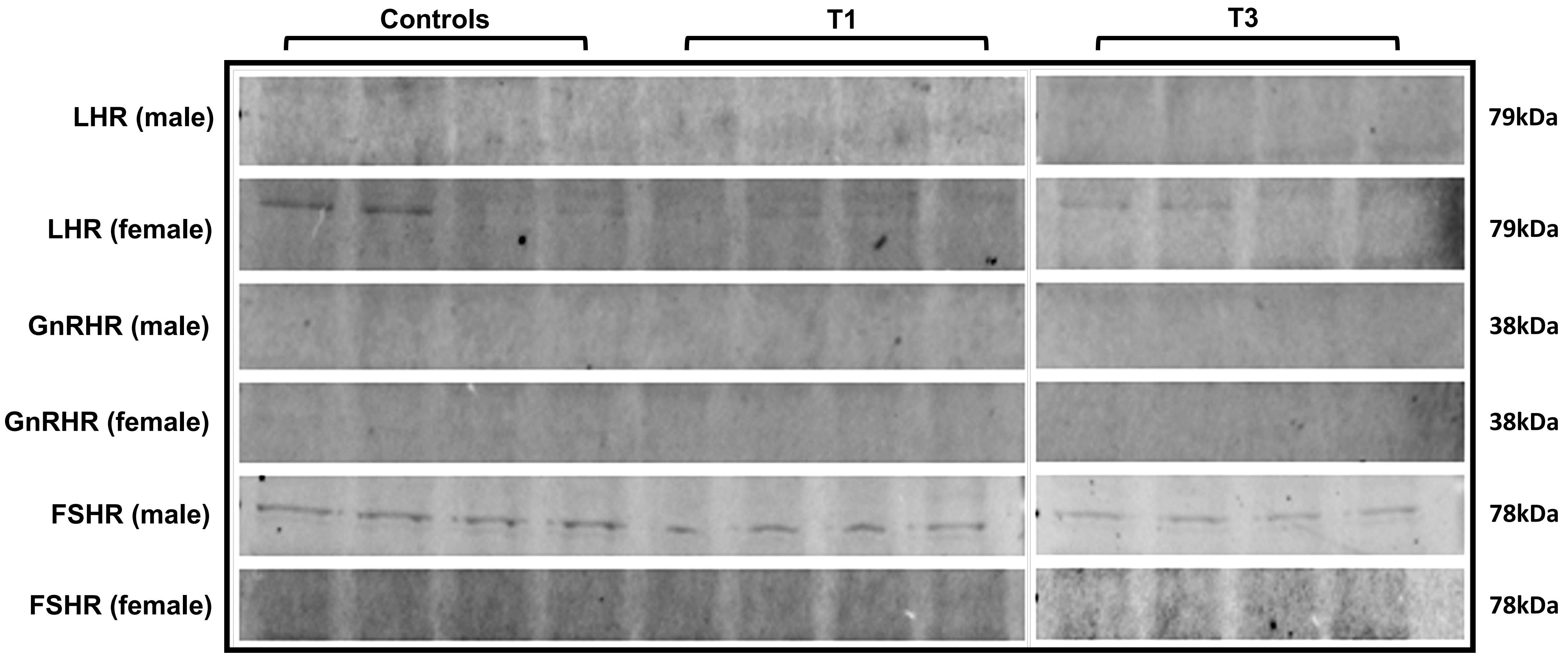

Supplemental Figure S3: Representative Western Blot images of LHR, GnRHR, and FSHR protein expression with control, T1, and T3 stimulation between HCF-Fs and HCF-Ms.

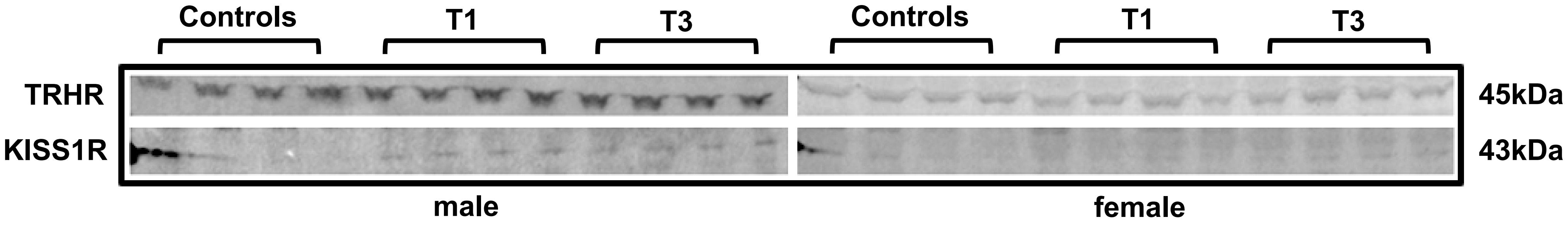

Supplemental Figure S4: Representative Western Blot images of TRHR and KISS1R protein expression with control, T1, and T3 stimulation between HCF-Fs and HCF-Ms.

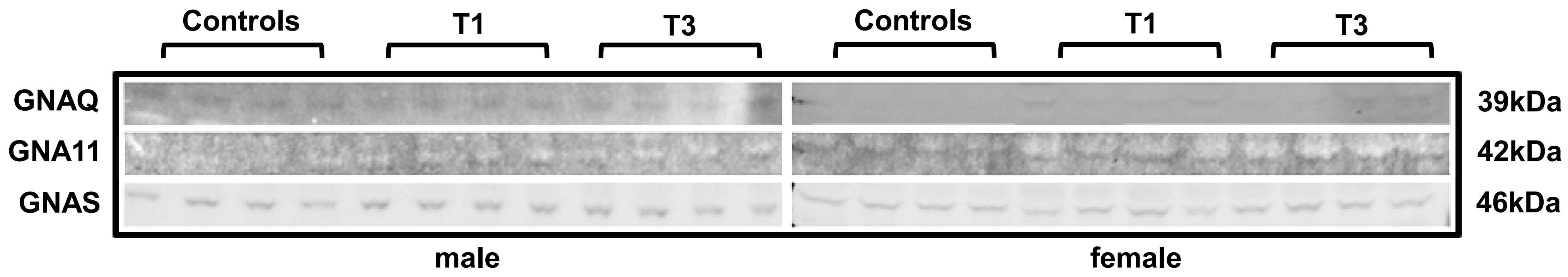

Supplemental Figure S5: Representative Western Blot images of GNAQ, GNA11, and GNAS protein expression with control, T1, and T3 stimulation between HCF-Fs and HCF-Ms.

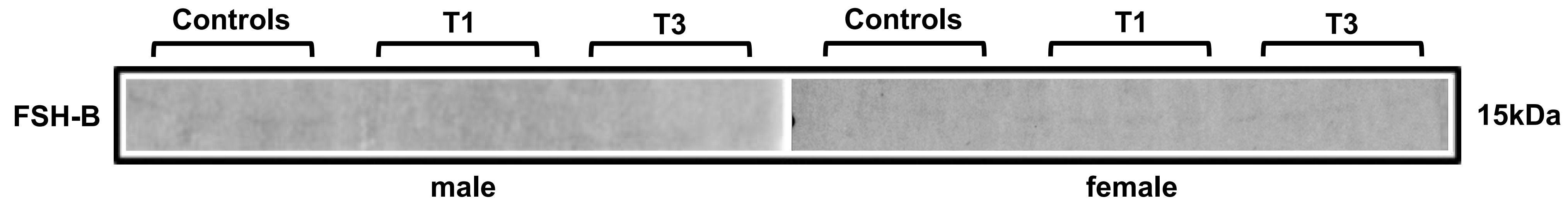

Supplemental Figure S6: Representative Western Blot images of FSH-B protein expression with control, T1, and T3 stimulation between HCF-Fs and HCF-Ms.

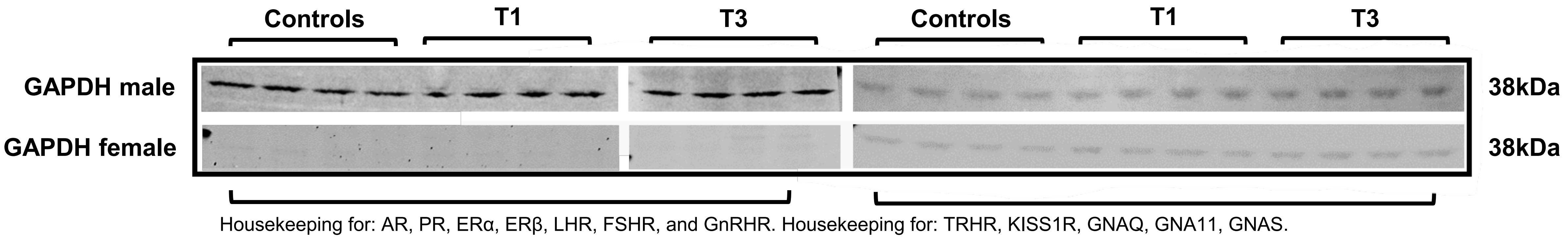

Supplemental Figure S7: Representative Western Blot images of GAPDH protein expression with control, T1, and T3 stimulation between HCF-Fs and HCF-
